# Application of HR-NMR for the Metabolic Kinetic Assessment of Red Mullet (*Mullus barbatus*) and Bogue (*Boops boops*) Samples during Different Temperature Storage

**DOI:** 10.3390/metabo13040482

**Published:** 2023-03-27

**Authors:** Alessandra Ciampa, Gianfranco Picone

**Affiliations:** 1 Department of Civil, Environmental, Land, Building and Chemical Engineering (DICATECH), Polytechnic University of Bari, Via E. Orabona n. 4, 70125 Bari, Italy; 2 Department of Agri-Food Sciences and Technologies (DISTAL), University of Bologna, Piazza Goidanich 60, 47521 Cesena, Italy

**Keywords:** bogue, chemometrics, fish freshness, high-resolution NMR (HR-NMR), metabolomics, red mullet, spoilage kinetic models, storage temperature

## Abstract

Fish freshness and quality can be measured through several indices that can be both chemical and physical. The storage temperature and the time that elapses following the catching of the fish are fundamental parameters that define and influence both the degree of freshness and nutritional quality. Moreover, they particularly effect the kind of fish we considered. In this research, it was observed how different temperatures of storage and shelf-life (+4 °C and 0 °C) may affect the metabolic profile of red mullet (*Mullus barbatus*) and bogue (*Boops boops*) fish samples over time, specifically observing the alteration of freshness and quality. In particular, a High-Resolution Nuclear Magnetic Resonance (HR-NMR)-based metabolomics approach was applied to study the metabolic profile changes that occur in fish spoilage. The HR-NMR spectroscopy data were useful for calculating a kinetic model that was able to predict the evolution of different compounds related to fish freshness, such as trimethylamine (TMA-N) and adenosine-5′-triphosphate (ATP) catabolites for the K-index. Furthermore, NMR in combination with chemometrics allowed us to estimate a further kinetic model able to represent the spoilage evolution by considering the entire metabolome. In this way, it was also possible to detect further biomarkers characterizing the freshness and quality status of both red mullets and bogues.

## 1. Introduction

High-quality standards are essential in all foodstuffs, and freshness in particular is a key determinant in consumers’ food choices, even if it remains an imprecisely defined concept [1]. The definition of freshness has always been a problem, as a comprehensive and objective measurement is lacking. The definition seems to be imprecise and difficult as it is strictly dependent on several factors that are related to a specific product and the quality of food sold, among other aspects such as price, store size, food safety, cleanliness, customer service, and brands sold [2].

When dealing with fish and fish products, a precise definition of freshness becomes very important, as among all foods they are much more perishable and, hence, susceptible to high postmortem changes that lead to different types of losses [3]. After a fish is captured, several changes, both metabolic and physical, occur, which translate into a high loss in its nutritional values. The deterioration of fish (fish spoilage) is the result of several phenomena that are both physic-chemical, biochemical, and microbiological, and begins at its postharvest, progresses rapidly after a few hours of landing [3], and continues all along the post-mortem phase [4]. It is also influenced by the method of fishing, the onboard handling, and storage temperature [5]. After catching, the rate of fish freshness is at the maximum level, but due to the action of bacteria, enzymes, and autoxidation of the fat, it progressively decreases over time. These transformations and alterations start affecting the metabolomic composition, which is also associated with physical characteristic changes, such as odor, muscle softness, and the color of the eyes and gills [3]. Hence, spoilage causes both nutritional value and organoleptic characteristic losses. Good handling practices on board the vessel should ensure that the fish retains its natural freshness to the maximum possible extent. Good onboard handling practices means good fish products, as the quality and acceptability of the end product depends on the quality of the raw material.

Metabolic transformation and alteration may be caused, above all, by limited refrigeration conditions such as storage at a temperature higher than 0 °C. The effect of temperature on the rate of fish spoilage has been largely demonstrated [6,7]. The rate of spoilage has been found to correspond to temperatures ranging from +1 to +2.5 °C, and spoilage at +5.5 °C occurs at twice the rate that it occurs at 0 °C. Fish should not be allowed to remain exposed to the sun on the deck (for a long time), which facilitates the rapid growth of microorganisms and accelerates enzymatic and bacterial spoilage. For this reason, the best method for the preservation of onboard fish freshness involves lowering its temperature below −1 °C with a rapid cooling that can be achieved through intimate contact between fish and small pieces of ice.

Commonly, the methods to evaluate fish freshness and quality may be divided into two categories: sensory and instrumental methods [8]. In the first case, sensory evaluation is defined as the scientific discipline used to evoke, measure, analyze, and interpret reactions to characteristics of food as perceived through the senses of sight, smell, taste, touch, and hearing [9]. Nowadays, in Europe, the most common method used for evaluating the freshness of fish and fish products in sensory analysis is the EU scheme [10] and the Quality Index Method (QIM) [11,12]. In the second case, the instrumental methods used to assess fish freshness are based on physical, chemical, and microbiological determination [13]. For example, the chemical methods try to define the limits of trimethylamine (TMA-N), total volatile basic nitrogen (TVB-N), formaldehyde, histamine, peroxides, and thiobarbituric acid content [14,15,16,17,18]. Each of these methods gives information only on a specific molecule that belongs to a specific class of compounds. Predictive microbiology has been suggested as a possible alternative to the traditional microbiological assessment of fish safety and quality [19]. The formation of these chemical indicators is the function of storage duration and temperature, and, thus, this function may be expressed in terms of a kinetic model. For example, Prabhakar et al. [20] developed a kinetic model for the formation of TVB-N and TMA-N in stored rohu fish (*Labeo rohita*) under limited refrigerated (+5 °C and 0 °C) and frozen (−5 °C) conditions. A kinetic model, in fact, can be used to predict the freshness and shelf life of fish and fish products [20]. The aim of this work was the development of a kinetic model to predict the formation of TMA-N and K-index in stored red mullet (*Mullus babrbatus*) and bogue (*Boops boops*) samples by using a high-resolution Nuclear Magnetic Resonance (HR-NMR)-based metabolomics approach [21]. This approach exploits the ability of HR-NMR to give information on the entire metabolic profile of fish samples via a single and rapid analysis, and, at the same time, avoid the use of hazard reagents [21,22,23]. Using the combination of this technique with chemometrics models, it has been possible to express the quality parameters from a holistic point of view, in which the entire metabolic profile competes in the definition of fish freshness and quality. The first part of this study focused on the evaluation of fish shelf-life through a kinetic model based on chemical indicators of freshness such as TMA-N and adenosine-5′-triphosphate (ATP) catabolites for the K-Index value. The concentration of TMA and the K-Index values have been calculated for both fish at 0 °C and +4 °C, over a period ranging from 0 to 11–15 days of shelf life. These data were obtained by applying a method already validated and published by Ciampa, Laghi, and Picone [8]. The obtained values have been compared to those foreseen by the EC Regulation N. 2077/05 that establishes, for TMA-N, a threshold of about 4–6 mg/100 g, while for K-Index, values ranging from 20% to 60% are considered within the range of acceptance for most fish species, with 60% being the limit for rejection [24,25].

Because spoilage is a multivariate natural phenomenon, as is the case for food deterioration, chemometrics tools could be useful to determine the shelf-life of food [26]. Thus, in the second part of this work, the unsupervised Principal Component Analysis (PCA) of NMR spectra has been employed for the evaluation of a multivariate kinetic model of the entire fish metabolic profile during storage at both 0 °C and +4 °C. PCA is one of the most popular multivariate techniques because it reduces the dimensionality, compresses the noise, and correlates measurements in a simple informational sub-space of the data set [27]. Moreover, it allows the identification of other metabolites closely related to fish freshness and takes into account storage conditions (time and temperature) [28].

## 2. Experimental Design

### 2.1. Samples Preparation and Storage Conditions

Fresh red mullets (*Mullus barbatus*) were caught in the Adriatic Sea (Cesenatico, Italy). Bogue (*Boops boops*) samples were purchased from Magna Grecia Mare—Portus Veneris (Leuca, Lecce, Italy). After catching, all fish were placed into polystyrene boxes, covered with ice flakes, and shipped immediately to the laboratory. Fish were individually inserted in open plastic pouches and stored at two different temperatures, +4 °C and 0 °C, to determine the effect on the generation of spoilage compounds in different conditions. In the first case, fish were placed inside a cold room (+4 °C) and the sampling was taken at 0, 1, 2, 3, 4, 7, 8, 9, 10, and 11 days after catching. For the second condition (0 °C), samples were put into a polystyrene box and covered daily with ice flakes (fish-to-ice ratio 2:1). Sampling was taken at 0, 1, 2, 3, 4, 7, 8, 9, 10, and 15 days after catching.

### 2.2. Sample Preparation for 1H-NMR Analysis

At each sampling time and temperature, a trichloroacetic acid extraction (TCA) was performed on three fish samples following the procedure set up by Boland [29] and adopted by Ciampa et al. [28]. An amount of 25 g of fish muscle was homogenized with 50 mL of 7.5 % trichloroacetic acid (TCA) water solution (1:2 *w*/*v*) and then filtered with filter paper (no. 4) from Whatman (Little Chalfont, Buckinghamshire HP7 9NA, UK). The pH of a 1 mL aliquot was adjusted to 7.80 ± 0.05 using 9 M KOH in an Eppendorf^®^ microfuge tube and centrifuged (Scilogex D30243, Rocky Hill, CT 06067, USA) at 14000 rpm and at +4 °C for 5 min to remove potassium trichloroacetate precipitate. The supernatant was stored at −80 °C until ^1^H-NMR measurements were performed.

### 2.3. ^1^H-NMR Measurements

A total of 72 samples for NMR analysis were prepared by adding to the thawed extractions 160 μL of a deuterated water (D_2_O) solution containing 6.25 mM of trimethylsilylpropanoic acid (TSP) as internal standard. All spectra were recorded at 298 K with a Bruker AVANCE spectrometer operating at a frequency of 600.13 MHz, equipped with an autosampler with 60 holders. Each spectrum was acquired using 32 K data points over a 7211.54 Hz spectral width and summing up 256 transients. A recycle delay of 5 s and a 90° pulse of 11.4 μs were set up. Acquisition time (2.27 s) and recycle delay were adjusted to be 5 times longer than the T1 of the protons under investigation, which has been considered to be no longer than 1.4 s. Saturation of residual water signal was achieved by irradiating it during the recycle delay at δ equal to 4.703 ppm. Each spectrum was processed with MestReC 4.9.8.0 (Mestreab Research SL, Santiago, Spain) by manually adjusting the phase and baseline and applying a line broadening factor of 0.5 Hz [30] (Figure S1). The peaks were assigned by comparing their chemical shift and multiplicity with the literature [22,23] (Table S3).

### 2.4. Data Processing and Principal Component Analysis (PCA)

After the spectra processing, the NMR data were saved as ASCII files for the subsequent statistical analysis using homemade algorithms written in the R program language (The R Project for Statistical Computing, version 2.4.0, Garr Mirror, Milan, Italy). The data processing follows the protocol described by Picone et al. [22]. Before statistical analysis, spectra were tidied by eliminating the interval region containing only the residual signal from the solvent (between 4.85 and 4.50 ppm) and those at both edges, which contain only noise (between 12.61 and 8.75 ppm, and between −0.55 and −3.38 ppm). A normalization preprocessing on the TSP signal at 0.00 ppm was applied to the entire matrix to minimize the differences caused by the sample extract dilutions. Before multivariate data analysis, the preprocessed matrix was condensed by subdividing each row into 150 bins, each integrating 120 data points (0.058 ppm). This practice is commonly used for HR-NMR data, both for reducing the number of total variables employed, which makes data mining faster, and for correcting for small peak misalignment problems, mainly due to slight pH differences that can significantly affect the peak chemical shift of pH-dependent molecules. To identify changes in metabolic profiles among samples in an unsupervised manner, principal components analysis of the preprocessed and mean-centered NMR data was performed using the R program language.

## 3. Results

**Figure 1:**
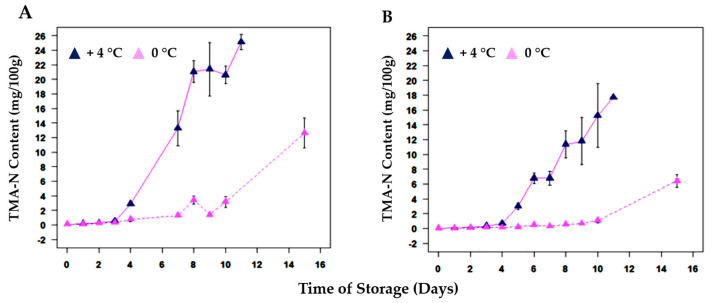
Kinetic of TMA-N concentrations (mg/100 g) for (**A**) red mullet (*Mullus barbatus*) and (**B**) bogue (*Boops boops*) samples during the storage at both +4 °C and 0 °C. All the concentrations were calculated via NMR by using the protocol fine-tuned by Ciampa et al. [8].

Figure 1 shows the kinetics of TMA-N formation over time for red mullets and bogue fish at both temperatures of storage. The TMA-N concentrations were determined by using the method demonstrated by Ciampa et al. [8] (Table S1). For samples at +4 °C, TMAO was converted into TMA-N after 3 days (T3) of storage. For samples at 0 °C, more days were required and TMA-N started to be detected after 10. The TMA-N trend over time reflects the kinetic model described by Howgate [31]. TMA-N formation showed an exponential increase in concentration followed by a dwell period when the TMA-N did not increase. For the author, the exponential phase can be mathematically modeled by the following Equation (1):

C_t_ = e^kt^ (1)

where C_t_ is the concentration at time t, k is the rate coefficient, and e is the base of natural logarithms. This expression gives a value of 1 at t = 0, and if C_t_ is expected to be 0 at t = 0, then 1 needs to be subtracted from the right-hand side or added to the left-hand [31]. The new expression is represented by the following Equation (2):

C_t_ = e^kt^ − 1 + a (2)

where a is a coefficient representing the concentration at t = 0.

**Figure 2:**
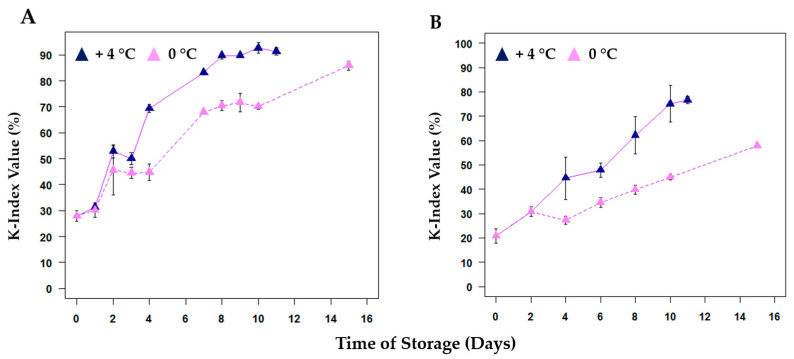
Kinetic of K-Index values (%) for (**A**) red mullet (*Mullus barbatus*) and (**B**) bogue (*Boops boops*) samples during the storage at both +4 °C and 0 °C. All the percentages were calculated via NMR by using the protocol fine-tuned by Ciampa et al. [8].

Red mullets stored at +4 °C showed a higher concentration of TMA-N after 11 days (T11) of storage than bogue samples stored under the same conditions. This can be explained by their different nutritional composition [32]. A significant role is played by the differences in the lipid/protein/water ratio during the degradation development: the higher this ratio, the slower the degradation of the fish [30]. In samples at 0 °C the degradation of TMAO in TMA-N is reduced, but still higher for red mullets. This variation is clear-cut and is due to the slower conversion of TMAO into TMA-N at 0 °C. In this condition, the conversion is replaced by a slow enzymatic reaction, which leads to the formation of dimethylamine (DMA) and formaldehyde [20,33]. Trimethylamine-N-oxide enzyme (TMAOase), concentrated in the internal organs and red muscles, has been known to catalyze the conversion of TMAO to DMA and formaldehyde [34]. The formaldehyde is not toxic itself, but may interact with amino acid residues, amino-terminal groups, and low molecular weight compounds, causing the denaturation and the “cross-linking” of proteins [35]. In addition to TMA-N, the K-Index values (Table S2) can also be used to create kinetic models for fish spoilage, and they are represented for both species in Figure 2. In the first days, the degradation is only due to the action of enzymatic spoilage, while at a later time, the increase in ATP catabolites, such as inosine (HxR) and hypoxanthine (Hx), is mainly due to the bacterial spoilage [36].

Hara and Uda [37] proposed a mathematical model relating K values to the rate of inosine monophosphate (IMP) breakdown by autolysis, based on a first-order reaction mechanism and according to the following Equation (3):

K_t_ = 100 − (100 − K_0_)e^−kt^ (3)

where K_t_ and K_0_ are K values at times t and the time when IMP is at its estimated maximum value, and k is the rate constant that can vary in function depending on the fish species and the storage [38].

**Figure 3:**
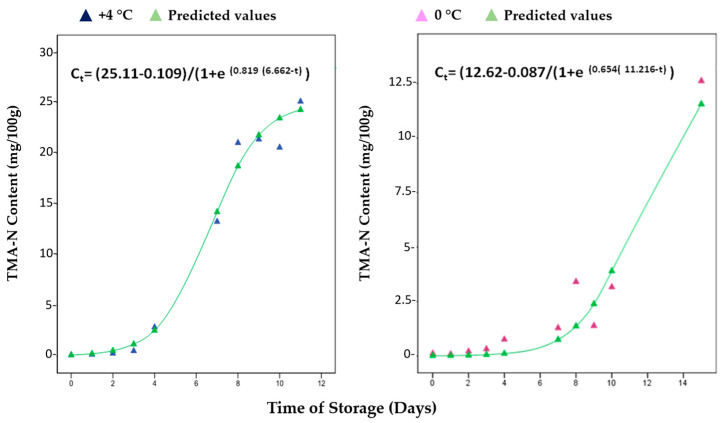
Fitted models of TMA-N concentrations (mg/100 g) for red mullets (*Mullus barbatus*) during the storage at both +4 °C and 0 °C. All the concentrations were calculated via NMR by using the protocol fine-tuned by Ciampa et al. [8].

**Figure 4:**
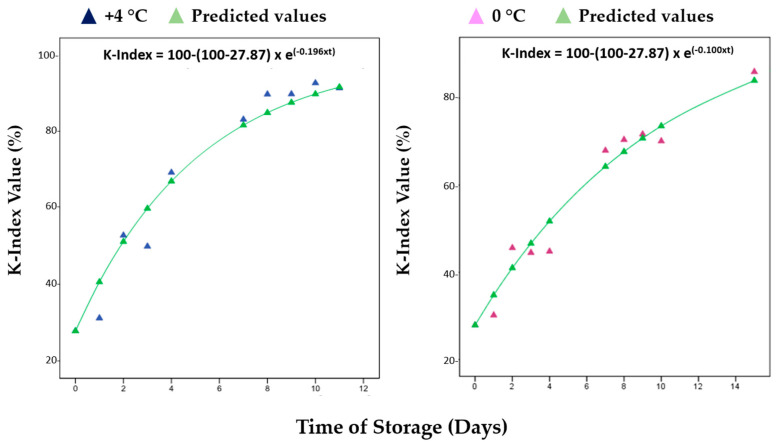
Fitted models of K-index (%) for red mullets (*Mullus barbatus*) during the storage at both +4 °C and 0 °C. All the percentages were calculated via NMR by using the protocol fine-tuned by Ciampa et al. [8].

The results obtained for the TMA-N and K-index from the storage study of red mullets (results for Bogue data are not available) were used to consider existing empirical models (kinetic) from the literature, such as those proposed by Howgate [31] and Hara and Uda [37] (Figure 3 and Figure 4). A logistic model, which is an advanced form of the exponential model [39], was found to have a good fit with the present experiment values. For TMA-N, a suitable model was the following Equation (4):

C_t_ = (C_max_ − C_min_)/(1 + e^K(T−t)^) (4)

where C_t_ is the TMA-N concentration at time t; C_min_ and C_max_ are the concentrations at the lower and upper asymptotes, respectively; K is the maximum growth rate, which occurs at the inflection point of the logistic curve; T is the time at the inflection point; e is the base of natural logarithms. Table 1 summarizes the values of the kinetic parameters for TMA-N at +4 °C and 0 °C by the exponential models plotted in Figure 3, in addition to the rate constants (k) for the K-Index in Figure 4.

The k(t) data demonstrate that fish spoilage at 0 °C occurs more slowly. Temperature plays a crucial role in altering the stability of seafood, as it controls the rate of bacterial spoilage and enzyme breakdown [40]: the higher the temperature, the faster fish spoil. Thus, the spoilage rate of fish may be reduced by good handling practices and effective temperature control from the catch. The immediate fish chilling after the catch and the fish’s storage at 0 °C through proper icing can reduce the rate of spoilage.

**Figure 5:**
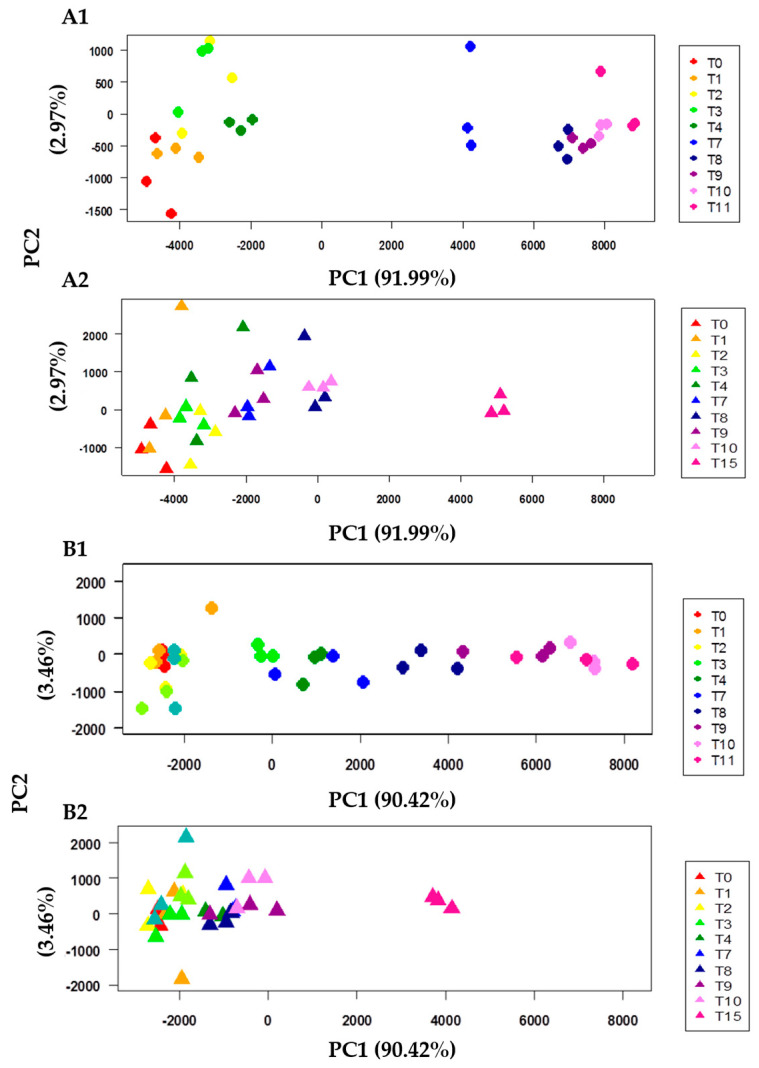
PCA scores plot of spectra data set. (**A1**) Red mullet (*Mullus barbatus*) and (**B1**) bogue (*Boops boops*) samples at +4 °C, and (**A2**) red mullet (*Mullus barbatus*) and (**B2**) bogue (*Boops boops*) samples at 0 °C. The PC models and graphs were calculated using the R program.

The content of TMA-N and the K-Index are high-quality evaluations for the determination of fish freshness, but they are related only to a specific metabolite (TMA-N) and/or pathway (ATP breakdown). The NMR-based metabolomic approach, on the contrary, allowed the assessment of fish freshness and the kinetic of spoilage by the analysis of the entire metabolome. A mathematical model describing the metabolome kinetic during storage was obtained by applying a chemometric analysis on the entire spectra data set. In particular, a Principal Component Analysis (PCA) was chosen as the predominant linear dimensionality reduction technique, and it has been widely applied to datasets in all scientific domains [27]. Moreover, PCA, beyond its ability to reduce the dimensionality, compresses the noise and correlates measurements in a simple informational sub-space of the data set [26]. Other supervised multivariate statistical analyses have also been developed, but they are mainly directed to highlight, within a complex mixture, those biomarkers indicating a clear difference among categories of samples [41]. For this reason, PCA has been largely employed as an exploration tool for the kinetic modeling of food quality, as demonstrated by Saavedra et al. [26]. In Figure 5, the PCA plot has been represented for red mullets and bogue at both temperatures of storage.

**Figure 6:**
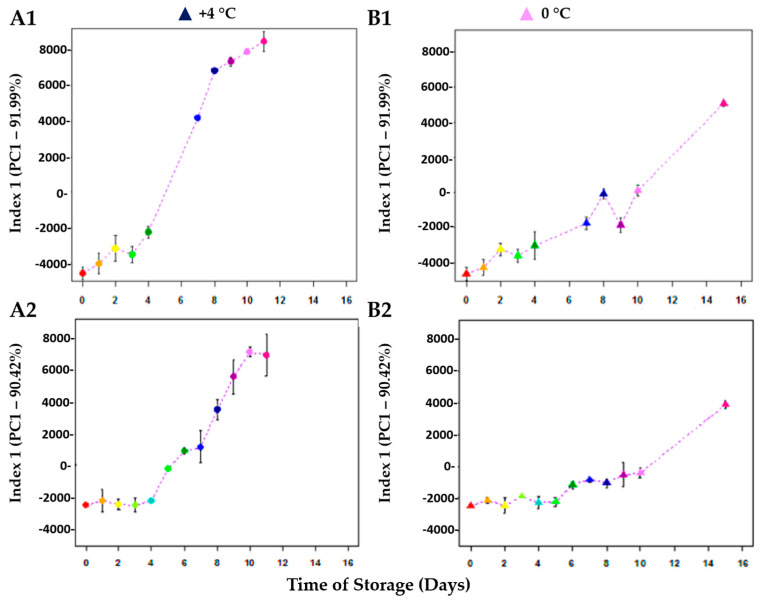
Fish metabolomic freshness kinetic curves were evaluated by reporting PC1 values vs. time of storage for red mullet (*Mullus barbatus*) samples at (**A1**) +4 °C and (**B1**) 0 °C, and bogue (*Boobps boops*) samples at (**A2**) +4 °C and (**B2**) 0 °C.

The PC1 axis represents the dimension along which the molecular composition of fish evolves during storage time. The effect of storage temperature is evident: while the samples at +4 °C are widely spread along PC1, more samples at 0 °C are gathered on the negative side of this component, except for samples at T15. The examination of PC1 loadings allows the identification of the metabolites responsible for the separation, highlighting the contribution of each variable in discriminating samples according to the time of storage. However, the source of variation is not solely confined to some metabolites, such as TMA-N or ATP catabolites. Rather, the whole metabolite’s profile is subject to change. Even nucleotides, some amino acids, and organic acids, such as formiate, lactate, and acetate, play an important role in the definition of fish freshness, as they are also involved in the formation, or lack thereof, of an agreeable odor [42], hence, giving information from a sensory point of view. In particular, the concentration of acidic (alanine, phenylalanine, tryptophan, glycine, methionine, valine, isoleucine, etc.) and basic (histidine and serine) free amino acids was generally found to increase and decrease during storage, respectively. These concentration changes were slow during the first days, as a consequence of protein autolysis, and at higher rates afterward, due to microbial development [28]. In summary, the PCA can give a unique score that is able to summarize all of the hundreds of parameters involved in the description of fish freshness, thus giving a measure of the molecular quality of fish. As PC1, or Index 1, is the main component describing the evolution of freshness over storage time, it can be taken to represent the summarizing freshness parameter, and can be described by the following Equation (5):

PC1 _molecular quality_ = α × par_1_ + β × par_2_ + γ × par_3_ + …..+ ω × par_nx_ (5)

where PC1 (Index 1) = TMA-N + K-Index + Free Amino Acids (FAA) + osmoregulators + organic acids, etc. (Figure S1 and Table S2). Figure 6 represents the kinetic curve obtained by plotting the PC1 values vs. the time of storage at both temperatures of storage.

These kinetic metabolomic curves, and, in particular, the one at +4 °C, reproduced a bacterial development curve [6] and, thus, they can be considered indexes of freshness. During the lag phase between 3–4 days, both fishes can be considered fresh. In this phase, the concentration of different compounds, such as amino acids, undergoes fluctuations that are appreciated by the consumers [28,43] from a sensory evaluation. Conversely, in the exponential multiplication phase, typically characterized by the development of off flavors, the red mullet fish may no longer be considered fresh.

**Figure 7:**
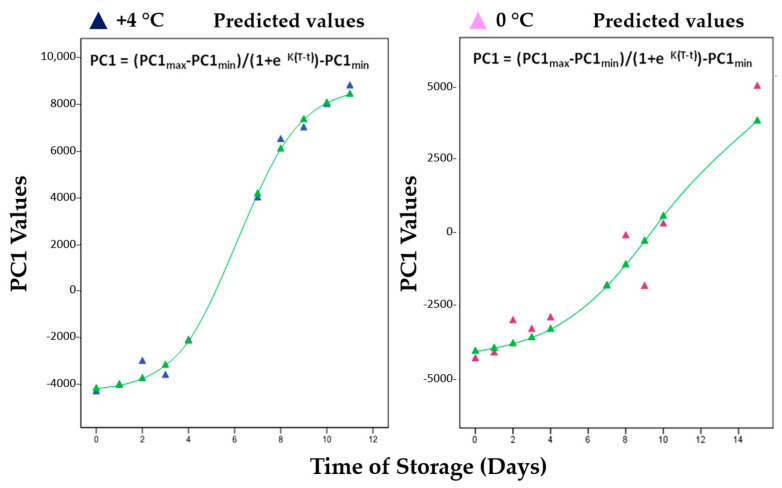
Fitted exponential and logistic models of PC1 values for red mullets (*Mullus barbatus*) during the storage at both +4 °C and 0 °C.

For the metabonomic curves of red mullets (those of Bogue data are not available), the following logistic model has been fitted, as shown in Equation (6):

PC1(t) = ((PC1_max_ – PC1_min_)/(1 + e^K(T − t)^) – PC1_min_ (6)

where PC1 (t) is the PC1 value at time t; PC1_min,_ and PC1_max_ are the values at the lower and upper asymptotes, respectively; K is the maximum growth rate, which occurs at the inflection point of the logistic curve; T is the time at the inflection point; e is the base of natural logarithms. In Table 2, the values of the kinetic parameters are summarized for PC1 at +4 °C and 0 °C by the exponential models plotted in Figure 7.

From the kinetic data of the metabolic profile, we also observe that the spoilage at +4 °C occurs faster for all compounds that, as extractable in a polar solvent, were examined by ^1^H-NMR. At 0 °C, the spoilage is slowed by 50% and these data confirm that the use of ice includes different functions, such as maintaining uniform low temperature and reducing autolysis and bacterial degradation.

## 4. Conclusions

In this study, a rapid method based on the NMR metabolomics combined with chemometrics has been applied to evaluate the evolution of fish spoilage during storage under different temperature conditions. Kinetic models, for shelf-life estimation, have been calculated for both classical freshness indicators, such as TMA-N content and the K-index. Using the PC1 values from the principal component analysis, it was possible to calculate univariate kinetic modeling that was able to evaluate fish spoilage by considering the entire fish metabolome. These metabolomic models are built up with the contribution of many compounds, including those that determine loss of freshness (TMA-N, HxR, and Hx), those that detect the nutritional status of fish (vitamins, amino acids, etc.), and those strictly related to sensorial aspects (for example, amino acids). Today, it is very important to set clear criteria to define the fish’s freshness and to predict its spoilage. In the future, the kinetic models by NMR could be a valid tool used to validate new methods designed for assessing fish freshness.

## Figures and Tables

**Table 1:** Kinetic parameters estimated from exponential models for TMA-N concentrations and K-Index values in red mullets (*Mullus barbatus*) at +4 °C and 0 °C.

Parameter (TMA-N)	+4 °C	0 °C	R^2^ (+4 °C) *	R^2^ (0 °C) *
k (days ^−1^)	0.819	0.654	0.984	0.941
T (days)	6.662	11.22	0.984	0.941
**Parameter (K-Index)**	**+4 °C**	**0 °C**	**R^2^ (+4 °C)**	**R^2^ (0 °C)**
k (days ^−1^)	0.196	0.100	0.960	0.962

* R^2^ is a regression error metric that justifies the performance of the model.

**Table 2:** Kinetic parameters estimated from exponential models for N-TMA concentrations and K-Index values in red mullets (*Mullus barbatus*) at +4 °C and 0 °C.

Parameter (PC1)	+4 °C	0 °C	R^2^ (+4 °C) *	R^2^ (0 °C) *
k (days ^−1^)	0.741	0.367	0.996	0.917
T (days)	6.185	9.749	0.996	0.917

* R^2^ is a regression error metric that justifies the performance of the model.

## Data Availability

Not applicable.

## Supplementary Materials

The following supporting information can be downloaded at: https://www.mdpi.com/article/10.3390/metabo13040482/s1. Figure S1: a typical 1H-NMR spectrum of fish recorded at 298 K with a Bruker AVANCE spectrometer operating at a frequency of 600.13 MHz. Table S1: TMA-N concentrations for red mullet (Mullus barbatus) and bogue (Boops boops) samples at T0, T4, T7, T11, and T15. All the concentrations were calculated via NMR by using the protocol fine-tuned by Ciampa et al. [8]. Table S2: K-index values (%) for red mullet (*Mullus barbatus*) and bogue (*Boops boops*) samples at T0, T4, T7, T11, and T15. All the concentrations were calculated via NMR by using the protocol fine-tuned by Ciampa et al. [8]. Table S3: metabolites identified in 1H-NMR spectrum, recorded with 600 MHz spectrometer, of bogue (*Boops boops*) muscle extract.
