# Supplementary material for: Application of HR-NMR for the Metabolic Kinetic Assessment of Red Mullet (Mullus barbatus) and Bogue (Boops boops) Samples during Different Temperature Storage
Source: Metabolites. 2023 Mar 27;13(4):482. doi: 10.3390/metabo13040482 (PMC10143401; doi:10.3390/metabo13040482)
Supplement: Supplementary file 1 [file metabolites-13-00482-s001.zip › metabolites-2273429-supplementary.pdf]

Supplementary Table S1. TMA-N concentrations for Red mullet (*Mullus barbatus*) and Bogue (*Boops boops*) samples at T0, T4, T7, T11, and T15. All the concentrations were calculated via NMR by using the protocol fine-tuned by Ciampa et al. [8].

	**TMA-N (mg/100g)**	
**Red mullet (*Mullus barbatus*)**	**+4 °C**	**0 °C**
T0	0.11 ± 0.03	0.11 ± 0.03
T4	2.856 ± 0.20	0.756 ± 0.30
T7	13.26 ± 2.36	1.285 ± 0.14
T11 / T15*	24.85 ± 1.07	12.62 ± 2.09
**Bogue (*Boops boops*)**		
T0	0.027 ± 0.0009	0.027 ± 0.0009
T4	0.657 ± 0.072	0.141 ± 0.030
T7	6.78 ± 0.679	0.304 ± 0.061
T11 / T15*	17.68 ± 4.31	6.46 ± 0.92

* T11 and T15 are the last days of sampling for storage at 0 °C and +4 °C respectively

Supplementary Table S2. K-index values (%) for Red mullet (*Mullus barbatus*) and Bogue (*Boops boops*) samples at T0, T4, T7, T11, and T15. All the concentrations were calculated via NMR by using the protocol fine-tuned by Ciampa et al. [8].

	**TMA-N (mg/100g)**	
**Red mullet (*Mullus barbatus*)**	**+4 °C**	**0 °C**
T0	27.87 ± 1.90	27.87 ± 1.90
T4	69.28 ± 1.50	44.78 ± 3.30
T7	89.74 ± 1.30	70.41 ± 1.90
T11 / T15*	92.67 ± 1.50	85.99 ± 1.80
**Bogue (*Boops boops*)**		
T0	10.42 ± 1.50	27.87 ± 1.90
T4	37.17 ± 2.60	35.06 ± 7.80
T7	60.64 ± 1.80	38.89 ± 2.40
T11 / T15*	79.45 ± 5.90	69.44 ± 2.40

* T11 and T15 are the last days of sampling for storage at 0 °C and +4 °C respectively

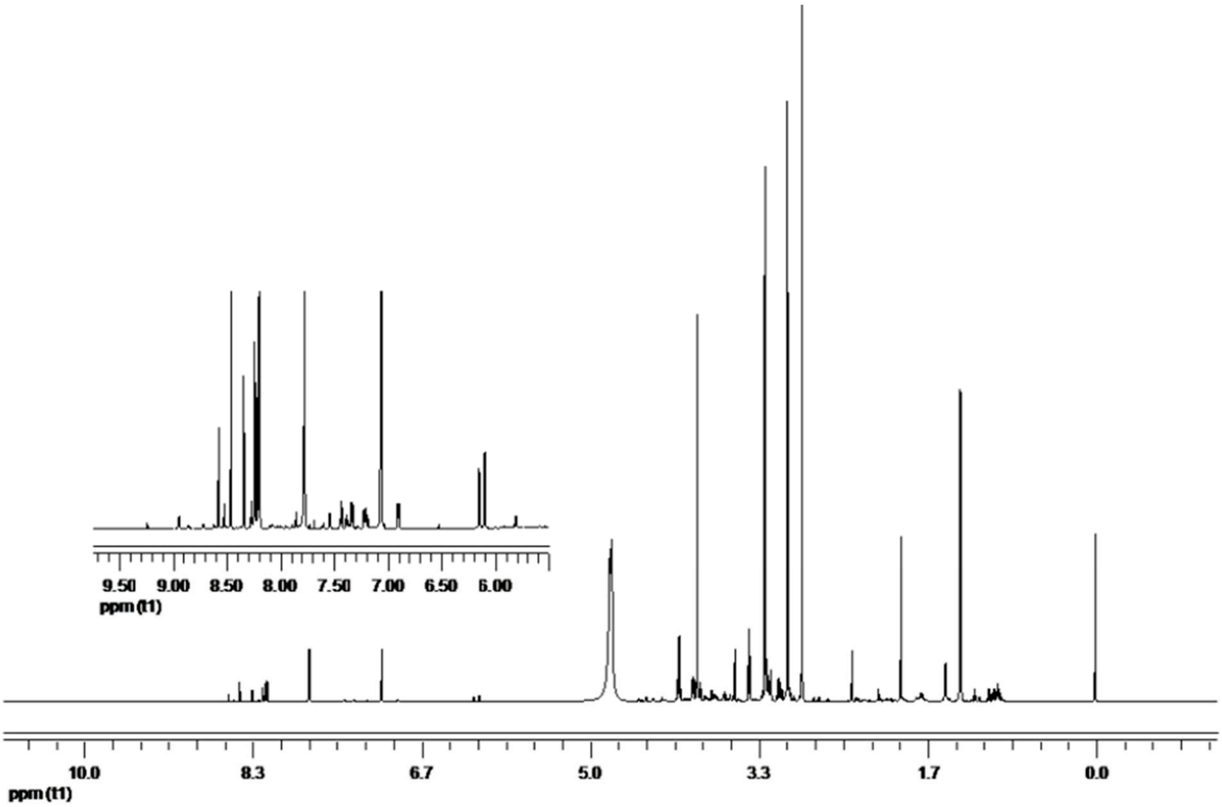

Supplementary Figure S1: a typical 1H-NMR spectrum of fish recorded at 298 K with a Bruker AVANCE spectrometer operating at a frequency of 600.13 MHz.

Supplementary Table S3 : metabolites identified in 1H-NMR spectrum, recorded with 600 MHz spectrometer, of Bogue (*Boops boops*) muscle extract.

Compound	Assignment	^{1}H (ppm)	Multiplicity
Isoleucine (Ile)	δ-CH_3	0.94	t
Leucine (Leu)	δ'-CH_3	0.96	d
Valine (Val)	γ-CH_3- γ′-CH_3	1.00-1.05	d
Ethanol	CH_3	1.19	t
Lactate (La)	β-CH_3	1.33	d
Alanine (Ala)	β-CH_3	1.49	d
Acetate	CH_3	1.93	s
Methionine (Met)	S-CH_3	2.14	s
Succinate	α, β-CH_2	2.41	s
Trimethylamine (TMA-N)	N-CH_3	2.90	s
Creatine/Phosphocreatine	N-CH_3 and N=C	3.04	s
Oxide Trimethylamine (TMAO)	N-CH_3	3.27	s
Taurin (Tau)	N-CH_2	3.42	t
Glycine (Gly)	α-CH	3.56	s
Glutamate (Glu)	α CH	3.75	t
Creatine/Phosphocreatine	N-CH_2	3.94	s
Serine (Ser)	β-CH	3.98	dd
α-Glucose (α-GLC)	CH-1	5.24	d
Inosine (HxR)	CH-1′, ribosio	6.10	d
Inosine 5′-monophosphate (IMP)	CH-1′, ribosio	6.14	d
Tyrosine (Tyr)	C3, 5H, ring	6.88	d
Histidine (His)	C2H ring/C4H ring	7.06/7.77	s
Tryptophan (Trp)	C5H ring	7.19	t
Phenylalanine (Phe)	CH-2,6	7.32	m
Hypoxanthine (Hx)	CH-8	8.19	s
Hypoxanthine (Hx)	CH-2	8.21	s
Inosine (HxR)	CH-8	8.233	s
Inosine 5′- monophosphate (IMP)	CH-8	8.236	s
Adenosine5′ triphosphate (ATP)	CH-8	8.27	s
Adenosine5′- diphosphate (ADP)			
Adenosine 5′-monophosphate (AMP)			
Inosine (HxR)	CH_2, ring	8.33	s
Formate (Fo)	CH	8.46	s
Inosine 5′- monophosphate (IMP)	CH_2, ring	8.57	s
